# How intrinsically disordered regions shape the function of CREB-binding protein

**DOI:** 10.1042/BST20250492

**Published:** 2026-04-30

**Authors:** Grace Gilbert, Daniel A. Bose

**Affiliations:** 1 Molecular and Cellular Biology, School of Biosciences, The University of Sheffield, Sheffield S10 2TN, United Kingdom.; 2 Nucleic Acids Institute, The University of Sheffield, Sheffield, S10 2TN, United Kingdom.; 3 Neuroscience Institute, The University of Sheffield, Sheffield S10 2TN, United Kingdom.

**Keywords:** acetylation, biomolecular condensates, CBP/p300, gene expression and regulation, IDRs, Intrinsically disordered proteins

## Abstract

CREB-binding protein (CBP) is a histone acetyltransferase and transcriptional co-activator that operates across the genome at *cis*-regulatory elements (CREs) to regulate gene expression. Comprising the majority of the protein, CBP has intrinsically disordered regions (IDRs) that separate its folded domains. Whilst previously regarded as passive linkers, active roles for these IDRs within CBP are beginning to be uncovered. Firstly, the flexibility afforded by these regions and the presence of binding motifs within them establish CBP as an interaction specialist that is able to interact with many binding partners at diverse CREs. In addition, the IDRs of CBP allow it to undergo liquid–liquid phase separation, forming condensates with emerging roles in transcription. Finally, an IDR within CBP performs an autoregulatory function that makes histone acetyltransferase activity sensitive to changing conditions within the cell. To build a comprehensive understanding of CBP function going forwards, it will be necessary to consider the contributions of the IDRs in addition to the structured domains of CBP and how these are integrated for the regulation of this key transcriptional protein.

## Introduction

Studies on protein function have predominantly focused on the structured, folded domains of a protein. However, it is increasingly being recognised that intrinsically disordered regions (IDRs)—regions that lack a stable tertiary structure—themselves can have functional roles. Proteins implicated in transcription and chromatin seem particularly overrepresented amongst intrinsically disordered proteins/IDR-containing proteins [1], suggesting that intrinsic disorder may be especially important for conferring functionality to chromatin-associated proteins [2].

**Figure 1:**
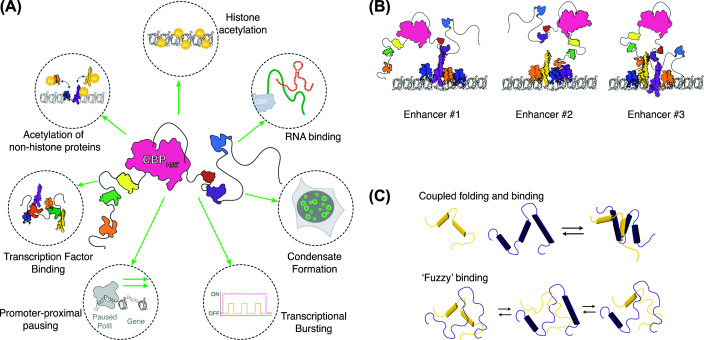
Different binding interactions are central to CBP’s function in gene expression (**A**) Different mechanisms used by CBP to regulate gene expression. (**B**) IDRs allow CBP to adopt distinct conformations, facilitating binding to unique combinations of transcription factors at different enhancers across the genome. (**C**) CBP–IDRs use a combination of mechanisms to mediate binding interactions with transcription factors. These include coupled folding and binding, where IDRs fold when interacting with a transcription factor, and ‘fuzzy binding’, where binding is maintained by many weak interactions, and structural elements are unstable and transient.

CREB-binding protein (CBP) is a large (∼265 kDa), multi-domain protein implicated in transcriptional regulation. The majority of CBP is intrinsically disordered (∼65%): the various folded domains with well-defined, stable structures and associated functions are separated by IDRs of variable length and composition [3]. CBP, along with its paralogue p300, functions as a transcriptional co-activator, operating by multiple mechanisms to influence transcription (Figure 1A). Many of these functions are interdependent, allowing CBP and p300 to integrate and respond differently to a vast array of signalling pathways and changes within the chromatin environment. Firstly, CBP functions as a histone acetyltransferase, acetylating key lysine residues within histone tails and on the nucleosome core. The negative charge of the acetyl groups neutralises the positive charge on lysine residues, decreasing their affinity for DNA and thus loosening the local chromatin structure to facilitate transcription, as well as acting as binding sites for histone ‘reader’ proteins [4]. CBP is also able to acetylate a range of non-histone proteins, such as transcription factors, which can alter properties such as their interactions with other proteins or DNA binding activity [4–6]. In addition, CBP has multiple protein interaction domains where transcription factors and other transcriptional cofactors are able to bind. This extensive binding ability allows CBP to perform a scaffolding function, localising key transcriptional proteins to promoter and enhancer regions and facilitating their interaction [7,8]. Consequently, at promoters where CBP is bound, CBP contributes to the recruitment, pausing, and release of RNA polymerase II (Pol II) [9–11]. CBP operates almost ubiquitously across the genome, with CBP- and p300-mediated H3K27ac being a recognised feature of active enhancers [12,13].

Although many of CBP’s functions can be ascribed to its structured domains, the IDRs within CBP also play key roles. Here, moving beyond the previously held assumption of IDRs as merely passive linkers, we explore the multiple ways in which the IDRs of CBP contribute to its function and regulation.

## The functional roles CBP IDRs

### CBP IDRs facilitate binding and establish CBP as an interaction specialist at *cis*-regulatory elements

CBP is well known for its ability to bind a wide variety of proteins; indeed, mass spectrometry has found over 1400 proteins interacting with CBP and p300 [14,15]. Although many of these interactions occur via the folded protein-binding domains of CBP, the flexibility afforded by the adjacent IDRs is important for this binding. Separation of stable folded domains by the flexible IDRs can overcome the issue of steric hindrance that may otherwise prevent CBP from achieving its multivalent interactions [16–18]. As a result, CBP is able to perform a scaffolding function at *cis*-regulatory elements (CREs), binding many different transcriptional regulators and facilitating their interaction at these sites. Importantly, the ability of IDR-containing proteins to adopt a range of different conformations and shift between these enables CBP to operate genome-wide at a huge proportion of CREs, each differing in the complement of proteins present and their relative spacing (Figure 1B) [3,19]. In this way, CBP—like other IDR-containing proteins—serves as an ‘interaction specialist’ [20], capable of mediating the diverse and complex binding inherent to transcriptional regulation.

As well as facilitating binding by the structured domains, IDRs themselves can serve as binding platforms for a range of proteins. These interactions are often associated with specific binding motifs: molecular recognition features and short linear motifs [2,21]. In some cases, IDRs will adopt a stable, folded conformation upon interaction with a binding partner, a process known as ‘synergistic folding’ or ‘coupled folding and binding’ (Figure 1C) [22,23], comprehensively reviewed in [3]. For example, the C-terminal NCBD domain of CBP is intrinsically disordered in its unbound state, but undergoes a disorder-to-order transition upon binding of transcription factors like NCOA3 [22]. In other cases, IDRs retain their disordered state even as they interact with other proteins, resulting in ‘fuzzy’ binding; for example, the transcription factor ZFP106 interacts with the IDR linking the KIX and bromodomain of CBP using ‘fuzzy’ interactions (Figure 1C) [24]. Together with the binding mediated by the structured domains, these diverse binding mechanisms enable CBP to scaffold the formation of large, multi-protein complexes required for transcription.

## An IDR within the catalytic domain of CBP performs an autoregulatory function

**Figure 2:**
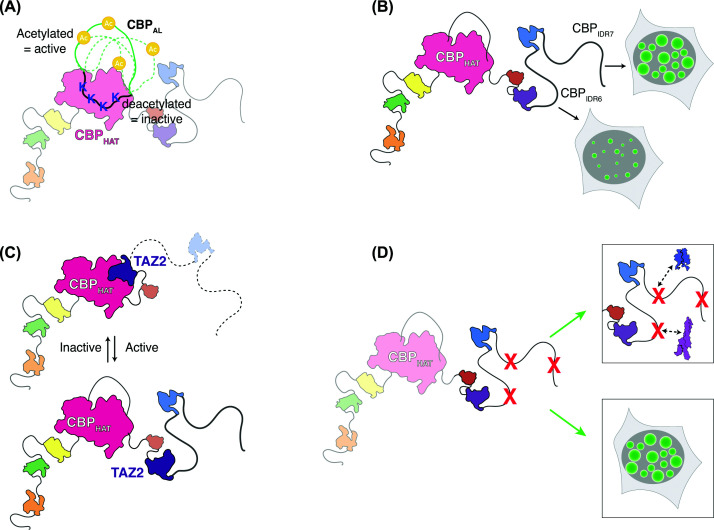
Roles of CBP IDRs in gene regulation and disease (**A**) Acetylation on the autoregulatory loop controls CBP KAT activity. (**B**) Two IDRs at the C-terminus of CBP positively and negatively regulate CBP condensate formation. (**C**) CBP–IDRs potentially control KAT activity by reducing inhibition by the TAZ2 domain. (**D**) Potential mechanism of disease variants in IDRs, include altered transcription factor binding and de-regulation of CBP condensates.

CBP–IDRs also play a critical role in autoregulating the catalytic lysine acetyltransferase (KAT) activity of CBP itself. Within the catalytic core of CBP lies a lysine-rich, disordered stretch of 63 amino acids referred to as the autoregulatory loop (AL) [25] or autoinhibitory loop in p300 [26]. The AL can bind intramolecularly to the active site of the KAT domain, blocking binding of acetylation substrates and thereby rendering CBP enzymatically inactive [26]. Like other IDRs, the disordered nature of the AL makes its residues accessible for post-translational modification, and enrichment for positively charged lysines facilitates binding to diverse RNAs [27], features that together allow IDR-containing proteins to serve as sensors for changes in the cellular environment [28]. Accordingly, the AL itself is a substrate for CBP and p300-dependent lysine acetylation, either via intramolecular or *in trans* autoacetylation; when acetylated, AL is displaced from the active site stimulating KAT activity (Figure 2A). Binding of eRNAs similarly promotes displacement of the AL and stimulation of KAT activity [27]. Notably, IDRs exert two levels of control on KAT activity, as autoacetylation *in trans* is promoted by recruitment of CBP and p300 to chromatin via interactions with transcription factors mediated by IDRs [29–31].

## Interaction of IDRs drives the formation of CBP-containing biomolecular condensates

Many IDR-containing proteins have been found to undergo liquid–liquid phase separation (LLPS), driven by homotypic and heterotypic interactions between their disordered domains [32] and often incorporating RNA [33]. The resulting condensates are regarded as membraneless organelles associated with key functional outputs, in which proteins and other biomolecules related to this function are concentrated relative to the dilute surrounding environment. Transcription is a complex and highly regulated process that must occur in a time- and cell type-specific manner for proper development and function. Over the last decade, the concept has emerged of transcriptional condensates acting as hubs in which transcription factors, transcriptional cofactors, and Pol II are concentrated together with target chromatin for efficient and tunable gene transcription [34–37].

CBP has long been known to exist in foci within the nucleus [38], and more recent studies investigating their properties support that these are biomolecular condensates formed by LLPS. For example, live-cell imaging has shown fusion and dispersion of these condensates over time, and fluorescence recovery after photobleaching and fluorescence loss in photobleaching show that CBP is highly mobile within the foci and can be rapidly exchanged with the dilute surrounding environment [29,39,40]. Critically, given the concentration-dependent nature of phase separation, CBP forms condensates at endogenous concentrations within the cell [40]. While there are additional *in vitro* biophysical criteria against which CBP condensates are yet to be assessed, observations within the nucleus are consistent with CBP undergoing phase separation to form liquid-like condensates. The enormous number of CBP-interacting transcription factors [15], the propensity of transcription factors to both form condensates [41] and interact with nuclear RNAs [42], and the ability of CBP itself to bind to RNA species at CREs [27,43,44] suggest a role for RNA in regulating CBP condensates. Consistently, treatment with RNase decreased the proportion of punctate nuclei and the size and intensity of condensates formed by CBP–GFP, supporting the idea of RNAs being an important component for their formation and/or maintenance [40].

The specific IDR(s) mediating phase separation can be studied by investigating the capability of isolated IDRs to drive condensate formation, and observing changes to condensate formation resulting from deletion of individual IDRs. Using these approaches, two neighbouring C-terminal IDRs within CBP have been identified as having opposing roles in its phase-separating behaviour [40]. Consistent with a condensate-promoting role, CBP_IDR7_ was sufficient for condensate formation in isolation utilising the OptoDroplet system, whilst deletion of CBP_IDR7_ prevented the formation of CBP–GFP condensates. In contrast, CBP_IDR6_ deletion yielded more condensates that had a greater size and intensity relative to expression of full-length CBP, suggesting that in the wildtype protein, CBP_IDR6_ operates to inhibit condensate formation. Interestingly, deletion of both CBP_IDR6_ and CBP_IDR7_ mimicked the effect of CBP_IDR7_ deletion, suggesting CBP_IDR7_ to be the most dominant of the two for promoting condensate formation. Work by others suggests that a ‘Goldilocks principle’ applies to condensate formation, in that the scale of condensate formation must occur in a certain range; otherwise, transcriptional output is affected [29,45–48]. This apparent balance between condensate-promoting CBP_IDR7_ versus condensate-inhibiting CBP_IDR6_ perhaps therefore reflects a mechanism that allows CBP condensate formation to be finely controlled for optimal function (Figure 2B). CBP_IDR6_ also seems to be important for determining the sensitivity of CBP condensate formation to changes in the cellular environment. When wildtype CBP was expressed, the proportion of nuclei with punctate CBP signal was increased by either global hyperacetylation or hypoacetylation, achieved using small molecule inhibitors. However, deletion of CBP_IDR6_ amplified the changes in condensate behaviour resulting from hyper/hypoacetylation, with dramatic changes in the number, size, and intensity of CBP condensates [40]. This suggests that CBP_IDR6_ may normally serve to buffer the scale of the response of CBP condensates to changing conditions within the cell.

The potential functional importance of CBP phase separation is supported by work on p300 showing that transcriptional bursting kinetics at a target locus differ depending on the ability of transcription factors for co-condensation with p300 [29]. With a transcription factor that co-condenses with p300, transcription of the target gene was rapid in onset and sustained. In contrast, with a transcription factor that fails to co-condense with p300—i.e. p300 foci did not assemble at the target site—transcription was slower to occur and less sustained. The same observations were made utilising genome-wide datasets: transcription initiation was faster and transcription sustained for longer when genes were enriched for p300 [29]. We note, however, that there is some contradicting evidence regarding the activation status of p300 within condensates [49] and whether condensate formation itself can alter transcriptional output [47], highlighting the challenging nature of studying phase separation in the context of transcription. Presumably a delicately balanced and highly regulated process, cautious interpretation is required with respect to *in vitro* studies of phase separation or studies utilising artificial systems within cells. Moving forwards, it will be important to clarify a functional role for CBP phase separation in transcriptional output by using novel approaches that enable CBP condensate behaviour to be studied in the nucleus at endogenous concentrations and at endogenous loci.

## Deletion of C-terminal IDRs of CBP alters the epigenetic landscape and transcriptional output

The importance of the IDRs within CBP for its function is highlighted by the dysregulation that occurs when these regions are deleted. Relative to expression of full-length CBP, expression of CBP_IDR6_- or CBP_IDR7_-deletion constructs altered genome-wide CBP binding and H3K27ac deposition [40]. Upon CBP_IDR6_ deletion, the number of CBP-binding sites increased genome-wide, and more CBP binding was observed at these sites, which correlated to an increase in the number and size of H3K27ac-enriched domains. In contrast, CBP_IDR7_ deletion resulted in an increased number of CBP-binding sites but decreased binding at these sites, together with a decrease in the number of H3K27ac-enriched domains. Consistent with these changes in CBP recruitment and H3K27ac deposition, deletion of either CBP–IDR results in an altered pattern of gene expression, with hundreds of genes differentially expressed relative to when full-length CBP is expressed.

The multifunctional nature of the IDRs within CBP complicates interpretation of experiments based on IDR deletion, as changes to any one—or a combination of these—may be driving altered functional output. IDR deletion also may compromise the function of the folded domains, further complicating interpretation. Indeed, an *in vitro* KAT assay—in which sample preparation presumably prevents condensate formation—suggested that intrinsic KAT activity is reduced by deletion of CBP_IDR6_ or CBP_IDR7_ [40]. Deletion of CBP_IDR6_ or CBP_IDR7_ could therefore potentially alter transcription via a direct effect on KAT activity. The result was reminiscent of work in p300 highlighting that the TAZ2 domain, adjacent to CBP_IDR6_, makes interactions with the KAT domain that inhibited KAT activity (Figure 2C) [50]. C-terminal CBP IDRs could relieve negative regulatory interactions between TAZ2 and CBP KAT, thus explaining their surprising ability to maintain CBP in an active state. Future work should focus on trying to unpick the exact mechanisms by which each of the IDRs in CBP contribute to normal transcriptional regulation.

## Exploring the relevance of CBP IDRs to disease

CBP has been implicated in a number of diseases, from cancer to the neurodevelopmental disorder Rubinstein-Taybi syndrome, in which heterozygous mutations in CBP/p300 lead to multisystem developmental abnormalities [51–55]. Given the importance of CBP for proper transcriptional output, it is unsurprising that altered CBP expression or function in either direction is associated with disease. However, with a primary focus of the impact of disease-associated mutations in CBP on KAT activity thus far, moving forwards it will be crucial to also consider altered protein–protein interactions and altered condensate formation as potential disease mechanisms, particularly in cases of mutations localising to CBP’s IDRs (Figure 2D) [56]. For example, we note that the neurodevelopmental disorder Menke-Hennekam syndrome is exclusively caused by mutations in the C-terminus of CBP/p300 [57–59], which we know to be critical for its phase-separating ability [40]. If there are disease contexts in which condensates are affected, correcting condensate formation, composition, distribution, or biophysical properties may prove to be a useful therapeutic avenue [48,60]. If CBP condensate formation is functionally important for transcription, manipulation of condensate formation may even be useful in disease states where transcriptional output is altered by an alternative mechanism. For example, global hypertranscription is a common feature of cancer: in such cases, perhaps transcription inhibition could be achieved by targeting CBP condensates rather than targeting mRNA synthesis directly [61]. Finally, the existence of CBP condensates within the cell may have implications for the delivery and action of therapeutic CBP/p300-targeting drugs, necessitating further investigation [62].

## Summary and perspectives

IDRs compose the majority of CBP, and appear to have multiple functional roles. Firstly, IDRs within CBP support its binding activity, affording flexibility that facilitates the multiple simultaneous interactions of the folded domains with their binding partners, and also containing binding motifs for protein interactions themselves. CBP IDRs also regulate the formation of CBP-containing biomolecular condensates, with neighbouring IDRs within the C-terminus seemingly having opposing roles in condensate formation. Finally, an IDR within CBP serves as an autoregulator of CBP KAT activity by binding intramolecularly to the catalytic core. Though examined in isolation, these functions are overlapping and interlinked; for example, acetylation status of the AL directly affects KAT activity, but also affects condensate formation [26,31,40]. Combined, the functions conferred by its IDRs establish CBP as a key player in transcription that is able to operate genome-wide to acetylate histones and other targets, and assemble the components of the transcriptional machinery. CBP is also able to ‘sense’ and respond to changes in the cellular environment: conformational changes and post-translational modifications of its IDRs can alter CBP interactions, KAT activity, and condensate formation, ensuring that transcriptional output is shaped by changes in the cellular context.

Going forwards, it will be important to characterise CBP phase separation further and clarify its functional relevance. It remains to be determined what other biomolecules are present within CBP-containing condensates and whether CBP is an active driver of condensate formation versus a client that is recruited to these. If we subscribe to the idea of CBP condensates acting as hubs of active transcription, we would expect to see co-localisation with components of the transcriptional machinery such as Pol II, nascent RNA transcripts, and CREs of target genes, as has been observed for p300. The precise mechanisms by which phase separation may influence transcription also remain to be uncovered. To do this will require moving beyond merely correlating condensate formation with transcriptional output, to dissect how factors such as CBP behave inside condensates themselves. Given the growing evidence for the involvement of RNAs in the formation and regulation of transcriptional condensates [33,63,64], we emphasise the importance of considering the contributions of RNA alongside the classic key players of DNA and proteins in further studies of CBP condensates in transcriptional regulation.

In summary, the IDRs of CBP complement the functional roles mediated by its structured domains and confer sensitivity of these functions to changing conditions within the cell. To build a comprehensive model of how CBP operates at CREs, future work must consider the contribution of its IDRs to CBP function and regulation.

## Perspectives

- IDRs are abundant amongst proteins implicated in transcription and chromatin, and disease-associated mutations often map to IDRs. IDRs comprise the majority of CBP and the altered CBP chromatin localisation, H3K27ac deposition and transcriptional output upon their deletion highlights their functional relevance.
- IDRs within CBP contribute to overall function by facilitating multivalent interactions, promoting the formation of biomolecular condensates within the nucleus and autoregulating catalytic activity.
- Future work should make use of novel approaches to further characterise the contribution of individual IDRs within CBP and clarify how these integrate with each other and the folded domains to establish CBP as a central protein in transcription regulation. In these studies, it will be important to maintain as far as possible normal physiological conditions to ensure the relevance of the developed model, particularly with reference to condensate formation.

## Open Access

Open access for this article was enabled by the participation of the University of Sheffield in an all-inclusive *Read & Publish* agreement with Portland Press and the Biochemical Society under an agreement with JISC.

## Abbreviations

AL: autoregulatory loop
CBP: CREB-binding protein
CREs: *cis*-regulatory elements
IDRs: intrinsically disordered regions
LLPS: liquid–liquid phase separation
Pol II: RNA polymerase II
KAT: lysine acetyltransferase
